# The rheology and interfacial properties of biomolecular condensates

**DOI:** 10.1007/s12551-025-01326-6

**Published:** 2025-06-30

**Authors:** Huan Wang, Zheng Shi

**Affiliations:** Chemistry and Chemical Biology, Rutgers University–New Brunswick, Piscataway, NJ 08854 USA

**Keywords:** Bimolecular Condensates, Phase Separation, Rheology, Viscoelastiity, Interfacial Tension

## Abstract

Biomolecular condensates are increasingly recognized as central regulators of numerous cellular processes. The bulk rheology of condensates (e.g., viscoelasticity) balances molecular mobility with structural stability, while the interfacial properties of condensates (e.g., interfacial tension) regulate condensate growth and their interactions with other cellular structures. Here, we review the functional roles of condensate rheology and interfacial properties, as well as diseases associated with their dysregulation. By summarizing emerging methodologies and quantitative measurements of condensate viscoelasticity and interfacial tension in the literature, we highlight key regulators of condensate material properties and discuss their implications in biology.

## Introduction

Biomolecular condensates have emerged as key organizational hubs that govern a myriad of cellular processes, including transcription, signaling, and stress responses (Banani et al. 2017; Shin and Brangwynne 2017). These condensates exhibit diverse material properties (namely, viscosity, elasticity, and interfacial tension), arising from the interplay of macromolecules inside or on the surface of the condensate. The material properties of condensates are critical determinants of their biological functions. Abnormal changes in these properties have been closely associated with aging-related diseases.

A central rheological property is viscoelasticity, which describes how a bulk material deforms or flows under external stress. Biomolecular condensates often display both fluid-like and solid-like behaviors (Alshareedah et al. 2024a; Patel et al. 2015; Shin and Brangwynne 2017). In the scaffold-client model, scaffold molecules (e.g., multivalent proteins) drive phase separation and establish a viscoelastic network via weak, multivalent interactions that allow transient bond formation (Mathieu et al. 2020). In contrast, client molecules are recruited to the condensate and can modulate the network’s material properties without being required for condensate formation (Banani et al. 2016; Ditlev et al., 2018). The liquid-like feature of condensates permits rapid molecular rearrangements, essential for dynamic processes such as transcriptional regulation (Riback et al. 2023), RNA processing (Wang et al. 2021a), and enzymatic activities (Testa et al. 2021). However, an overly fluid condensate may fail to maintain a stable structure, whereas an excessively rigid condensate may hinder essential dynamics or the clearance of malfunctioning molecules (Yamasaki et al. 2020), or form pathological aggregates (Lin et al. 2015; Patel et al. 2015).

The surface of a condensate is mechanically characterized by its interfacial tension, which compartmentalizes individual condensates from their environment. Interfacial tension governs the nucleation, ripening, and fusion of condensates; determines the miscibility between condensates; and regulates the wetting between condensates and other cellular structures such as membranes, cytoskeletal filaments, DNAs/RNAs, or other condensates (Gouveia et al. 2022).

Several comprehensive reviews have discussed the material properties of biomolecular condensates, providing detailed insights into the emergence and quantification of their rheological and interfacial characteristics (Gouveia et al. 2022; Pappu et al. 2023; Wang et al. 2022b; Zhou et al. 2024). Here, we focus on reviewing the quantitative values of condensate material properties, factors that modulate them, and their functional relevance in cell biology.

## The viscoelasticity of biomolecular condensates

Viscoelastic materials exhibit both viscous (fluid-like) and elastic (solid-like) behaviors under external mechanical force. Physically, it reflects a material’s ability to dissipate energy (viscosity) while also storing and recovering energy (elasticity) under stress (Serra-Aguila et al. 2019).

The mechanical response of a material is often dependent on the rate of the applied force. Under ultra-high frequency stress (> 10^9^ Hz), even water could display elastic-dominated responses (O'Sullivan et al. 2019). In contrast, materials traditionally considered as solids can flow at long observation times (Edgeworth et al. 1984). The viscoelasticity of a condensate determines its deformation, marked as strain (ε), under external stresses ($\sigma$) Two types of symbolling systems are commonly used to describe the viscoelasticity of condensates: viscosity (*η*) and elasticity (*E*) dictate a condensate’s steady-state response under mechanical stress, whereas the loss modulus (*G”*) and the storage modulus (*G’*) are more commonly used to describe the relation between oscillating stress and strain (Barnes et al. 1989; Jawerth et al. 2020). The mechanical properties of condensates at different time scales can uncover distinct physical information. At ultrafast timescales (~ nanoseconds), the mechanical response of a condensate reflects molecular interactions and chain vibrations (Jaross, 2016, 2020; Neshasteh et al. 2025). At slower timescales, condensate mechanics can be relevant for mechanotransduction (milliseconds), cargo trafficking (seconds to minutes), cell migration (minutes to hours), and cell division (hours) (Aiken and Holzbaur, 2021; Ghusinga et al. 2016; Hoffman et al. 2011; Milo and Phillips, 2015; Park et al. 2023; Zhao et al. 2024). Investigators should therefore choose the observation timescale that best matches the biological process they wish to study.

Several linear viscoelastic models have been shown to effectively describe the rheology of condensates, including the Maxwell model (Jawerth et al. 2020), the Kelvin-Voigt model (Alshareedah et al. 2024a; Cheng et al. 2025), and the Jefferys model (Wang et al. 2024; Zhou, H., 2021) (Table 1). The Maxwell model (a spring and a dashpot in series) captures how a material flows under stress yet can exhibit transient elasticity, which is particularly useful for condensates that display fluid-like behavior at long timescale. In contrast, the Kelvin-Voigt model (a spring and a dashpot in parallel) enables a representation where elastic and viscous forces act in parallel but eventually reach an elastically dictated equilibrium. Adding another dashpot to the forementioned two-element models results in two variations (the Maxwell-form and the Kelvin-form) of the Jeffreys model (Table 1). These two forms are mathematically equivalent: setting ${\eta }_{1}^{\text{M}}=\frac{{\eta }_{1}^{\text{K}}\cdot {\eta }_{2}^{\text{K}}}{{\eta }_{1}^{\text{K}}+{\eta }_{2}^{\text{K}}}$, ${\eta }_{2}^{\text{M}}=\frac{{\left({\eta }_{1}^{\text{K}}\right)}^{2}}{{\eta }_{1}^{\text{K}}+{\eta }_{2}^{\text{K}}}$, and ${E}^{\text{M}}={\left(\frac{{\eta }_{1}^{\text{K}}}{{\eta }_{1}^{\text{K}}+{\eta }_{2}^{\text{K}}}\right)}^{2}{E}^{\text{K}}$ convert the Maxwell-form to the Kelvin-form. However, the Kelvin form is more convenient when analyzing strain response to a stepwise stress (Guevorkian et al. 2010), whereas the Maxwell form is more commonly used in frequency-dependent measurements of the viscoelastic moduli (Zhou 2021). Compared to the Maxwell model, the Jeffreys model allows condensates with zero elasticity but finite viscosity and can better fit the response of viscoelastic fluids where multiple relaxation timescales are observed. Notably, when a viscoelastic material approaches the ideal limits of a purely viscous liquid or a purely elastic solid, the Maxwell and Kelvin-Voigt models give unphysical results: in the pure liquid limit, the Maxwell model predicts an infinite elastic modulus, while in the pure solid limit, the Kelvin–Voigt model approaches a viscosity of zero. In other words, the Maxwell model does not allow materials with zero elasticity (and finite viscosity), whereas the Kelvin-Voigt model does not allow materials with materials with infinite viscosity (and finite elasticity). To describe the viscoelasticity of more complex condensates, linear models with more elements or nonlinear models may be needed.

**Table 1:**
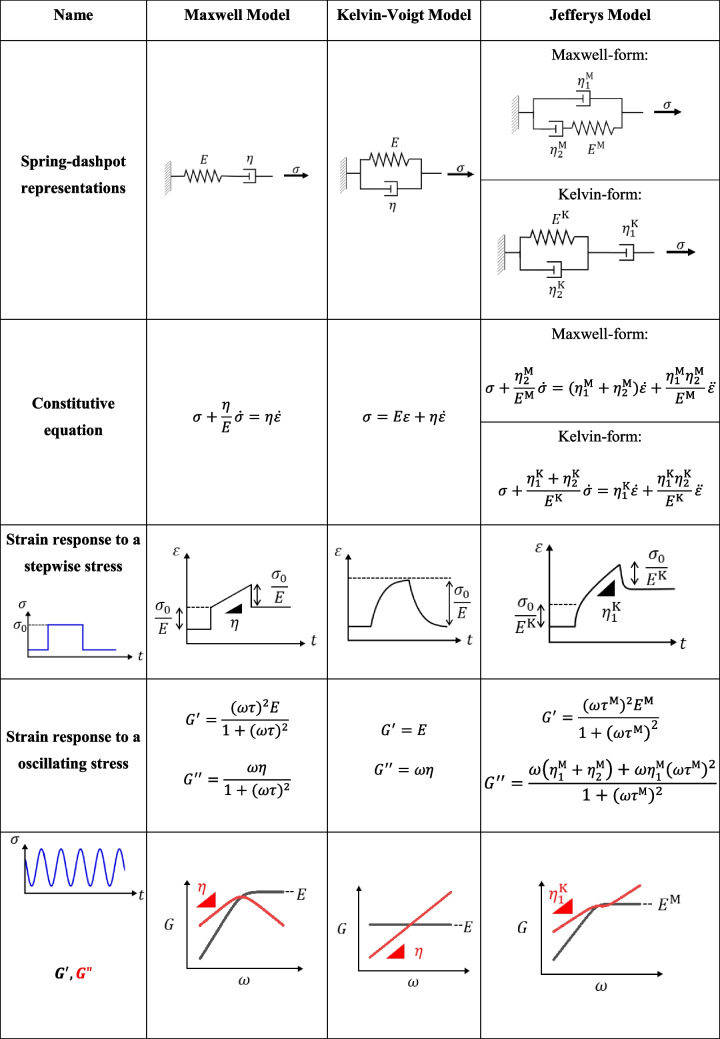
Three commonly used viscoelastic models to describe condensate rheology

$\tau = \frac{\eta }{E}$*;* ${\tau }^{M}= \frac{{\eta }_{2}^{M}}{{E}^{M}}$*.* Triangles in the graph indicate the slope (in linear scales) of the corresponding regions of the curve

Various techniques have been developed to probe the viscoelasticity of condensates (Table 2) (Alshareedah et al. 2021a). Broadly, these methods measure viscoelasticity in either a passive or active manner. Passive viscoelasticity quantification relies on tracking the diffusion of tracers within the condensate. Assumptions regarding the size and shape of the tracers (typically via the Stokes–Einstein relation) need to be implemented to infer condensate viscoelasticity. Examples of such techniques include fluorescence recovery after photobleaching (FRAP) (Feric et al. 2016; Murakami et al. 2015; Taylor et al. 2019) and single-particle or single-molecule tracking (Elbaum-Garfinkle et al. 2015; Taylor et al. 2016). These techniques, especially FRAP, are widely used due to their relative technical simplicity. However, FRAP has been criticized for its limited quantitative accuracy when applied to a 3-dimensional condensate, as boundary conditions (e.g., bleaching profile outside the imaging plane, exchange at the condensate interface) are often unknown (Taylor et al. 2019). Within a limited observation window, FRAP is often unable to distinguish a highly viscous Newtonian liquid (i.e., slow but full recovery) from a viscoelastic solid (i.e., relatively fast but partial recovery). Meanwhile, single-particle tracking, albeit more quantitative, often requires delicate chemical modifications of the particle surface to achieve condensate incorporation while avoiding the aggregation of incorporated particles. There are several other passive rheology techniques that are beginning to be adapted to the condensate field. Fluorescence correlation spectroscopy (FCS) (Beutel et al. 2019) reports the local diffusion of molecules within condensates. Molecular rotors have been developed to probe the nanoscale viscosity of the condensate through the rotors’ fluorescence lifetime or spectrum (Paez‐Perez and Kuimova, 2024; Ye et al. 2021). Brillouin microscopy is a label-free optical method that can be used to quantify condensate viscoelasticity. The light scattered by a solid-like material typically has a higher Brillouin frequency shift and narrower line width compared to that of a liquid-like material, allowing 3-D mechanical mapping in live cells (Bevilacqua et al. 2020).

**Table 2:** Literature values for the interfacial tension (γ), viscosity ($\eta$), and elasticity (E) of biomolecular condensates. Data in this table are used to plot Fig. 1 and Fig. 2

Protein name	γ (μN/m)	${\varvec{\eta}}$(Pa s)	*E* (Pa)	Method	Reference
P granules	1	1		FRAP ($\eta$) and Fusion ($\gamma /\eta$)	Brangwynne et al. (2009)
LAF-1(125 mM NaCl)	280	34		FRAP ($\eta$) and Fusion ($\gamma /\eta$)	Elbaum-Garfinkle et al. (2015)
LAF-1 + 5 μM RNA (125 mM NaCl)	100	12.8			
LAF-1(250 mM NaCl)		14			
LAF-1(400 mM NaCl)		8			
Whi3 (25 μM Whi3, 60 mM KCl)	0.15	6		Particle tracking ($\eta$) and Fusion ($\gamma /\eta$)	Zhang et al. (2015)
Whi3 + RNA BNI1 (8 μM Whi3, 53 nM RNA,150 mM KCl)	2	19			
Whi3 + RNA CLN3 (8 μM Whi3, 53 nM RNA,150 mM KCl)	2	28			
NPM1_in vitro_	0.8	0.74		FRAP ($\eta$) and Fusion ($\gamma /\eta$)	Feric et al. (2016)
NPM1_in vivo_	0.4	37			
FIB1_in vitro_	2.5	100			
Nucleolus	1.5	3000		Surface fluctuation ($\gamma$) and Fusion ($\gamma /\eta$)	Caragine et al. (2018)
PGL-3 (75 mM KCl)	5	0.7	14*	Active Microrheology by optical tweezer	Jawerth et al. (2018)
PGL-3 (115 mM KCl)	3	0.5	2*		
PGL-3 (150 mM KCl)	2	0.15	0.5*		
PGL-3 (180 mM KCl)	1	0.06	0.2*		
FUS	3.1	0.7	0.35	Active Microrheology by optical tweezer	Jawerth et al. (2020)
PGL-3 (75 mM KCl, *t* = 0.5 h)	4.5	4.4	56		
PGL-3 (75 mM KCl, *t* = 1.5 h)	19.3	39	50.7		
PGL-3 (75 mM KCl, *t* = 45 h)		10000	6	Particle tracking	
FUS (*t* = 6 h)		50	0.1		
polyK	17	0.204		Particle tracking ($\eta$) and Fusion ($\gamma /\eta$)	Fisher and Elbaum-Garfinkle (2020)
polyR	100	14.4			
FoxA1	0.04–0.28			Optical tweezer force measurement	Quail et al. (2021)
LAF-1 RGG	210	10	10^#^	Micropipette aspiration	Roggeveen et al. (2023); Wang et al. (2021b)
Lipid droplet	40,000	0.1		FRAP ($\eta$) and Micropipette aspiration ($\gamma$)	Chorlay et al. (2021); Ivanovska et al. (2023)
Lipid droplet + lipid monolayer	1000	0.1			Chorlay and Thiam (2018)
Endocytic Puncta	70	350	59	Shape analysis ($\gamma$, *E*), relative FRAP to cytoplasm ($\eta$)	Bergeron-Sandoval et al. (2021)
Cytoplasm		0.35	43.5	Active Microrheology by optical tweezer ($\eta ,E$)	
[RGRGG]_5_-dT40 (25 mM NaCl)	1600	5.5		Particle tracking ($\eta$) and Fusion ($\gamma /\eta$)	Alshareedah et al. (2021c)
[RGRGG]_5_-dT40 (100 mM NaCl)	1250	4			
[RGRGG]_5_-dT40 (325 mM NaCl)	700	1.6			
[RGRGG]_5_-dT40 (425 mM NaCl)	440	1			
SH3 + lysozyme (0.15 M KCl)	106	10.1	66.43*	Active Microrheology by optical tweezer	Ghosh et al. (2021)
SH3 + proline-rich motif (0.15 M KCl)	73.4	3.75	42.88*		
Proline-rich motif + heparin (0.15 M KCl)	67	0.53	4.82*		
polyK + heparin (1 M KCl)	57.1	0.3	3.43*		
[RGRGG]_5_-rU40		4	10*	Particle tracking ($\eta ,E$)	Alshareedah et al. (2021b)
[RGFGG]_5_-rU40		10	20*		
[RGYGG]_5_-rU40		20	70*		
[RGSGG]_5_-rU40		0.4	0.2*		
[RGPGG]_5_-rU40		0.19	0.1*		
[RGPGG]_5_-dT40		0.13	0.03*		
[RGPGG]_5_-G5T30C5		2	2*		
HA-Protamine (0 M NaCl)	31.9	0.3052	0.1*	Particle tracking ($\eta$) and Fusion ($\gamma /\eta$)	Hong et al. (2022)
HA-Protamine (4 M NaCl)	4200	28.5	2*		
MAP65		4.7–9.5		FRAP	Sahu et al. (2023)
Glycinin (50 mM NaCl)	125	5000	2000*	Active Microrheology by optical tweezer ($\eta ,E$) and Fusion ($\gamma /\eta$)	Mangiarotti et al. (2023)
Glycinin (100 mM NaCl)	120	1200	4000*		
Glycinin (150 mM NaCl)	15	200	1000*		
E-K sequences	284–2840	3.17–47.9		Molecular dynamic simulations	Sundaravadivelu Devarajan et al. (2024)
LAF1 sequences	98–496	3.37–23.44			
DDX4 sequences	75–599	1.9–28			
FFssFF	96	856		Active Microrheology by optical tweezer ($\gamma$), and Particle tracking ($\eta$)	Zhang et al. (2024a)
LLssLL	109	123			
MMssMM	38	0.22			
ELF3 PLD	49	24		FRAP-ID	Santamaria et al. (2024)
Synapsin + α-synuclein (median)	240	5000	10,000	Micropipette aspiration with whole-cell patch clamp	Wang et al. (2025)
Synapsin + α-synuclein (low αSyn partitioning)		20	500^#^		
Synapsin + α-synuclein (high αSyn partitioning)		1,000,000^##^	1,000,000^##^		
Synapsin + 3% PEG	23	25	10^#^	Micropipette aspiration	
Synapsin IDR + 10% PEG	69	12.9	10^#^		
Synapsin + 10% PEG	100	110	10^#^		
Synapsin + 3% PEG + 1 mg/mL cytosol (*t* = 23 h)		474	2000		
Synapsin + 3% PEG + 3 μM α-synuclein	2	50	10^#^		
Synapsin + 3% PEG + 3 μM α-synuclein + 1 mg/mL cytosol (*t* = 21 h)		150,000	5000		
Synapsin + 3% PEG + 9 μM α-synuclein	40	750	10^#^		
Synapsin + 3% PEG + 23 nM SVs	5	70	10^#^		
Synapsin + 3% PEG + 46 nM SVs	3	2,000	10^#^		
hnRNP A1-LCD (wild type)		2.57	1.494*	Particle tracking ($\eta$, $E$)	Alshareedah et al. (2024a)
allF		0.69	0.107*		
allY		1.10	2.764*		
W-		3.08	2.633*		
YtoW		6.79	1.542*		
FtoW		12.76	11.23*		
allW		73.50	29.57*		
FFssFF	96	856		Active Microrheology by optical tweezer ($\gamma$), and Particle tracking ($\eta$)	Zhang et al. (2024a)
LLssLL	109	123			
MMssMM	38	0.22			
SARS-CoV-2 N protein		11	30*	Particle tracking ($\eta ,E$) and Micropipette aspiration ($\eta$)	Favetta et al. (2024)
Phosphorylation N protein (phosN)		4	1*		
N + polyrA		64	150*		
phosN + polyrA		12	10*		
N + 1-1000RNA		192	300*		
phosN + 1-1000RNA		59	100*		
N + N-RNA		430	1000		
phosN + N-RNA		230	1000		
polyK + heparin (0.5 M KCl)		700	45,000*	Scanning probe microscopy	Naghilou et al. (2025)
polyK + heparin (0.85 M KCl)		8	20*		
polyK + heparin (0.9 M KCl)		5	10*		
polyK + heparin (1 M KCl)		5	10*		
polyK + heparin (1.1 M KCl)		2	5*		
Nucleolar GC	1.6	43**	0.125^#^	Micropipette aspiration	Cheng et al. (2025)
Nucleolar DFC	0.6^#^	230**	2.7		

^#^: estimated upper bound; ^##^: estimated lower bound

*: G’ at 10 Hz; **: apparent viscosity

**Fig. 1:**
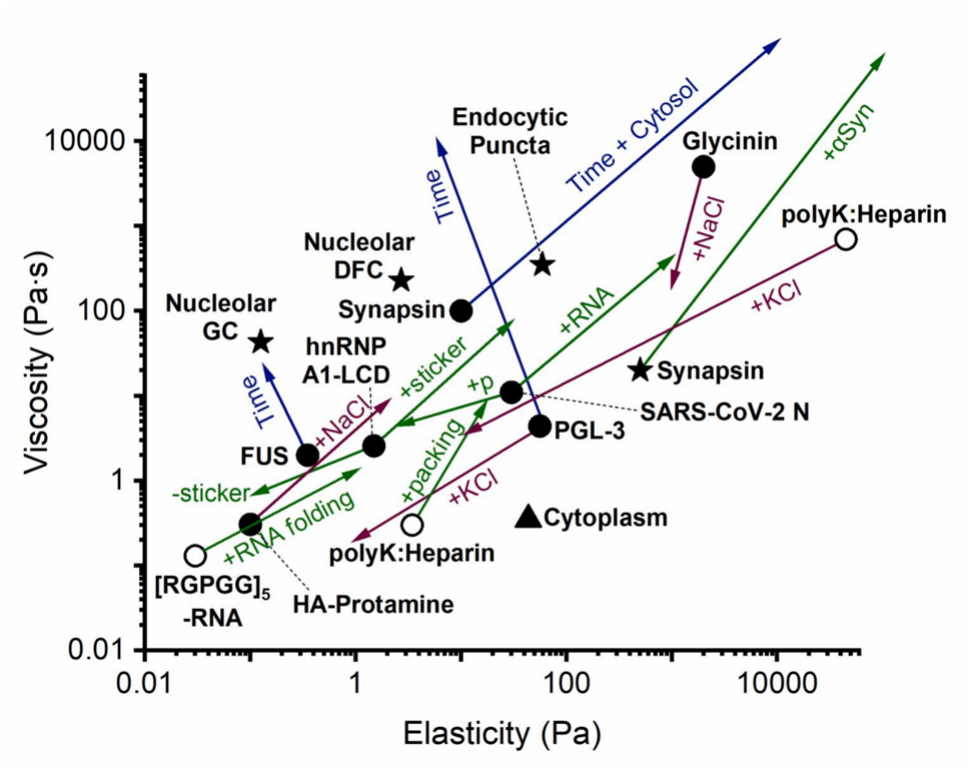
Plot of condensate viscosity vs. condensate elasticity. Markers: open circle, engineered protein condensates; closed circle, purified biological protein condensates; star, biological condensates in living systems; triangle, cytoplasm. Arrows: green, biological regulators; purple, regulators related to the aqueous environment; blue: “aging” effect

**Fig. 2:**
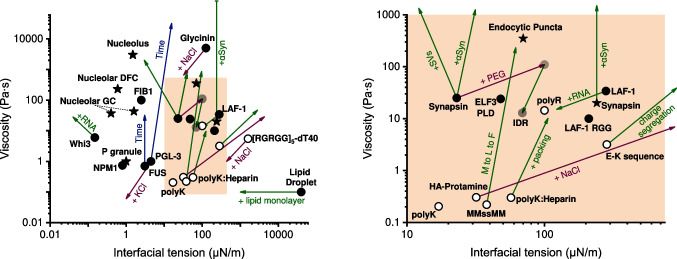
Plot of condensate viscosity vs. condensate interfacial tension. The shaded region in the upper plot is zoomed-in on the right. Markers: open circle, engineered protein condensates; closed circle, purified biological protein condensates; star, biological condensates in living systems. Arrows: green, biological regulators; purple, regulators related to the aqueous environment; blue: “aging” effect

Active viscoelasticity can be probed by applying controlled forces to a condensate and quantifying the resulting deformations, using techniques such as micropipette aspiration (MPA) (Roggeveen et al. 2023; Wang et al. 2021b), atomic force microscopy (AFM) (Li, X. et al. 2023; Santamaria et al. 2024), or optical tweezers (Alshareedah et al. 2024a; Català-Castro et al. 2025; Sundaravadivelu Devarajan et al. 2024). Although specialized instrumentation is often necessary, these methods enable richer and more quantitative characterization of condensate rheology. Additionally, fluorescent labels are not required in active rheology techniques, avoiding potential artifacts caused by fluorescent labeling and excitation (Barkley et al. 2024; Ibrahim et al. 2024). In addition to externally applied mechanical perturbations, the capillary force that drives the coalescence of condensates can be exploited to probe condensate viscoelasticity (Eggers et al. 1999; Ghosh and Zhou 2020; Ghosh et al. 2021; Sahu and Ross 2023). When two condensates encounter, they relax into a single sphere to minimize interfacial area. The characteristic fusion speed (condensate length divided by fusion time) is governed by the ratio of condensate interfacial tension ($\gamma$) to their viscosity ($\eta$). With a separate measure of interfacial tension, condensate viscosity can be inferred from the fusion measurement. Moreover, Strom et al. developed an optogenetic tool called VECTOR that is directly applicable in vivo. VECTOR utilizes condensate fusion to generate local capillary forces, revealing quantitative insights into chromatin viscoelasticity (Strom et al. 2024). Molecular dynamics simulations can track the motions of individual proteins and their chains, through which the viscosity of condensates can be calculated either from diffusion or from molecular motion under externally applied shear stress (Sundaravadivelu Devarajan et al. 2024; Tejedor et al. 2023). Interfacial tension can be extracted from the simulated stress tensor using the Kirkwood–Buff relation (Sundaravadivelu Devarajan et al. 2024).

The viscoelasticity of condensates can be strongly affected by the length scale of the measurement. For the same condensate, bulk viscosity of the condensate at the micrometer scale is typically thousands of times greater than that of water (Table 2), whereas nanoscale molecules within the condensate may experience an environment similar to a dilute aqueous solution (Galvanetto et al. 2023). By measuring the viscosity of ProTα and H1 at different length scales of observation, Galvanetto et al. proposed that the high macroscopic viscosity arises from a network of multivalent interactions among oppositely charged proteins. At the molecular level, however, these proteins rapidly reorganize, forming transient contacts that are short-lived compared to chain reconfiguration. Such scale-dependent viscosity enables the condensate to maintain structural stability while still allowing rapid molecular exchange in condensates and across its interface (Hoffmann et al. 2023). Beyond the differences imposed by the observational length scales, proteins themselves can assemble into condensates of different sizes (~ 10 nm to 10 μm) (Das, T. et al.; Demmerle et al. 2023; Forman-Kay et al. 2022). If the internal molecular structure remains unchanged, the viscoelastic properties of condensates should likewise be independent of their size.

### Viscoelasticity of condensates is an important regulator of biological functions

#### Physiological

Biomolecular condensates rely on a balance of fluid-like viscosity and solid-like elasticity to regulate essential cellular processes. In living cells, condensates can exhibit a continuum of soft-matter states between “pure liquid” and “pure solid” such as glassy liquids, viscoelastic gels, and plastic solids, distinguished by their (sometimes nonlinear and time-dependent) viscoelasticity (Alberti and Hyman, 2021; Català-Castro et al. 2025; Feric et al. 2015; Frey and Görlich 2007; Jawerth et al. 2020; Linsenmeier et al. 2022; Naghilou et al. 2025; Tejedor et al. 2023). These material properties regulate molecular mobility and structural stability within condensates, enable condensates to function as hubs for transcription (Bose et al. 2022), RNA processing (Wang et al. 2021a), and maintaining adaptability to environmental changes (Banani et al. 2017). If a condensate were purely viscous with a low viscosity, molecular components would diffuse freely but might not sufficiently resist deformation to maintain organizational features under stress. For instance, during early Drosophila development, the success in regulating the physical properties of P bodies can support the transition from a matured oocyte into an early embryo (Sankaranarayanan et al. 2021), while the liquid-to-solid transition of oskar ribonucleoprotein granules is essential for oskar localization, translation, and development (Bose et al. 2022). Conversely, if a condensate were purely elastic or solid-like, molecular mobility would be arrested, which could hinder dynamic processes such as enzymatic reactions (Testa et al. 2021), disassembly or clearance of aggregation-prone condensates (Yamasaki et al. 2020), gene activation and expression (Fu et al. 2025). During the growth of microtubules within microtubule-associated protein condensates, the gel-like condensates limit tubulin diffusion. As a result, tubulin is confined to the surface layer of the condensate, ultimately affecting the growth rate of microtubule asters (Sahu et al. 2023). Viscoelasticity is also important in the process of autophagy. Larger forces are needed to deform highly viscoelastic condensates, thereby hindering selective autophagy (Yamasaki et al. 2020).

The intracellular spaces, such as the cytoskeleton-rich cytoplasm or chromatin-dense nucleoplasm, are mechanically active (Meng et al. 2024; Rosowski et al. 2020). The viscoelasticity of condensates determines their responsiveness to intracellular forces. For instance, Rashid et al. demonstrated that external forces mobilize chromatin and nucleoplasmic proteins, leading to condensate dissolution (Rashid et al. 2023). Gerlitz et al. proposed that chromatin condensation is required for cell migration, highlighting a functional link between mechanical adaptation of condensates and cellular dynamics (Gerlitz and Bustin 2010). Zhao et al. revealed that nucleoli undergo dynamic fission and fusion processes, which are regulated by the viscoelasticity of condensates as well as the mechanical properties of their surrounding environment (Zhao et al. 2024). During mitotic exit, the pericentriolar material needs to adopt a gel-like structure to avoid disassembly under pulling forces from the microtubule (Enos et al. 2018; Mittasch et al. 2020). Bergeron-Sandoval et al. proposed that endocytic puncta are viscoelastic, allowing them to store mechanical stress when wetting the membrane and thus enable membrane remodeling during actin-independent endocytosis (Bergeron-Sandoval et al. 2021). Additionally, several condensates have been reported to contain mechano-responsive domains that can undergo conformational changes under intracellular forces (Bustin and Misteli 2016; Cheng and Case 2023; Dupont and Wickström, 2022; Negri et al. 2023).

#### Pathological

Many protein condensates are found to be metastable and tend to undergo an irreversible liquid-to-solid transition (Alshareedah et al. 2024a; Das et al. 2024b; Jawerth et al. 2020). This dysregulation of condensate viscoelasticity is increasingly recognized in pathological transitions such as neurodegeneration, transcriptional dysfunction, and immune evasion (Mathieu et al. 2020).

- Neurodegeneration

Extensive evidence has shown that the aberrant liquid-to-solid transition of proteins, such as FUS, α-synuclein, hnRNPA1, Tau, TDP-43, are linked to neurodegenerative diseases (Alberti and Dormann 2019; Choi et al. 2024; Dewey et al. 2012; Gui et al. 2023; Kanaan et al. 2020; Lin et al. 2021; Molliex et al. 2015; Patel et al. 2015; Ray et al. 2020). Those solidified condensates often are resistant to cellular clearance, leading to their accumulation over time. Moreover, by sequestering various proteins and RNA molecules, they can disrupt intracellular stress responses and signaling pathways, leading to widespread damage within neurons.

Amyotrophic lateral sclerosis (ALS)/frontotemporal dementia (FTD): Mutations in FUS and TDP-43 are closely associated with ALS and rare cases of FTD (Mackenzie, Ian RA et al. 2010). Wild-type TDP-43 and FUS condensates exhibit reversible liquid-like behavior in cells. However, disease-associated mutations enhance molecular attraction, leading to condensates with higher viscosity (Gopal et al. 2017; Patel et al. 2015) that can impair axonal transport. Moreover, mutations in the LCD of TIA1 were found to slow down the dynamics of stress granules (SGs) and affect neuronal activity (Mackenzie et al. 2017). Beyond mutation-driven liquid-to-solid transitions, post-translational modifications can also increase condensate viscosity and contribute to ALS and FTD (Hofweber and Dormann 2019). For instance, the loss of arginine methylation in FUS increases the viscosity of FUS condensate both in vitro and in cells, aligning with findings that unmethylated and monomethylated FUS accumulates in the brains of patients with FTD (Hofweber et al. 2018).

Tauopathies and Alzheimer’s disease (AD): Under physiological conditions, Tau plays a critical role in maintaining axonal structure by stabilizing microtubules. In tauopathies, however, hyperphosphorylation of Tau causes it to detach from microtubules and adopt pathological conformations that culminate in the formation of amyloid fibrils (Iqbal et al. 2016). Boyko et al. revealed that the disease-associated mutations, ΔΚ280 and P301L, did not measurably alter tau’s propensity to phase separate. However, these mutations accelerated the liquid-to-solid transition of tau condensates (Boyko et al. 2020).

Huntington’s disease: Huntington’s disease (HD), a fatal neurodegenerative disorder, arises from pathological expansions of a polyglutamine (polyQ) tract in exon 1 of the huntingtin protein (HTT). HTT_ex1_ variants with various polyQ lengths can undergo liquid-liquid phase separation (LLPS) to form condensates in yeast and mammalian cells; however, only those containing more than 42 glutamines exhibit a time-dependent liquid-to-solid transition, forming irreversible, solid-like assemblies (Peskett et al. 2018). This rheological shift aligns with the genetic threshold for HD pathogenesis, where polyQ tracts ≥ 42 residues invariably trigger disease (Finkbeiner 2011).

(b) Cancer

While much attention to condensate viscoelasticity has been directed toward neurodegenerative diseases, the pathological consequences of aberrant condensate viscoelasticity are also emerging in cancer. Many oncogenes and tumor suppressors, including transcription factors and RNA-binding proteins, form condensates that influence gene expression programs essential for cell proliferation, apoptosis, and metastasis (Bouchard et al. 2018; Tong et al. 2022). For instance, transcriptional coactivators can concentrate within condensates to drive oncogenic signaling and modulate the expression of growth-related genes (Banani et al. 2017). Under normal conditions, these condensates maintain sufficient fluidity to allow rapid turnover of co-activators and co-repressors, thereby enabling finely tuned transcriptional control. However, disease-related mutations that disrupt the dynamics of condensates can compromise transcription. For example, substituting Tyr with Phe in the a-kinase–anchoring protein AKAP95 markedly slows its diffusivity within condensates, ultimately impairing its role in splice regulation (Li et al. 2020).

(c) Viral infection

Growing evidence suggests that condensates may play a role in infectious diseases (Wang et al. 2021a). Pathogens can exploit condensates to enhance their propagation, while host cells utilize condensates to detect pathogens and trigger defense mechanisms. In this context, viral condensates typically maintain a high fluidity to facilitate rapid replication and dissemination (Etibor et al. 2021; Glon et al. 2024; Nikolic et al. 2017; Zhou et al. 2019). Intriguingly, drugs that lead to the hardening of viral condensates are able to inhibit respiratory syncytial virus (RSV) replication in the lungs of infected mice (Risso-Ballester et al. 2021). Meanwhile, phosphorylation of the SARS-CoV-2 nucleocapsid protein—which is known to regulate the viral life cycle—reduces the viscoelasticity of the condensate (Carlson et al. 2020; Favetta et al. 2024).

### Factors that regulate the viscoelasticity of condensate

The viscoelasticity of the biomolecular condensate emerges from a complex interplay of weak, multivalent interactions that are directly encoded in biomolecular sequences and highly sensitive to environmental conditions and molecular modifications (Pappu et al. 2023; Zhou, H. et al. 2024). Therefore, it is crucial to understand how biomolecular sequences and factors such as pH, salt concentration, post-translational modifications, temperature, and crowding reagents influence the viscoelasticity of condensates (Fig. 1). In principle, the viscosity and elasticity of a material can be regulated independently (Chaudhuri et al. 2016); however, as shown in Fig. 1, existing measurements on the viscosity and elasticity of biomolecular condensates suggest that factors that increase the viscosity of condensates tend to also increase their elasticity (with the exception of the aging effect on FUS and PGL-3 condensates).

a Sequence features

Sequence of a protein encodes the molecular grammar that drives phase separation and directly regulate the viscoelasticity of formed condensates. Several studies have revealed the relations between protein sequence and condensate viscoelasticity (Alshareedah et al. 2021b, 2024a; Rekhi et al. 2024; Sundaravadivelu Devarajan et al. 2024). Alshareedah et al. reported that the viscoelasticity of hnRNP A1 low-complexity domain (A1-LCD) is dependent on the strength of the aromatic residues (referred to as the stickers), with decreasing strength from Trp to Tyr and to Phe. Replacing all Phe with Trp increased the viscosity of A1-LCD from ~ 0.7 to ~ 70 Pa s, and elastic modulus (at 10 Hz) from 0.1 to 30 Pa, as shown in Fig. 1. Meanwhile, mutation of Gly to Ser (referred to as the spacers) triggered rapid aging of the A1-LCD condensate.

The effect of point mutations can vary significantly between proteins. For example, Rekhi et al. observed that Gly-to-Ala mutation had minimal effects on the rheology of artificial intrinsically disordered proteins. In contrast, Gly-to-Ala mutation significantly increases the viscosity of FUS condensates (Wang, Jie et al. 2018). Similarly, Niaki et al. found Gly mutations (G187S, G225V, and G399V) or Arg mutations (R216C, R244C, R514G, R521C, and R521G) of FUS result in significant decreases in condensate viscosity (Niaki et al. 2020).

In a simple polymer solution, the viscosity of the solution typically increases with the length of the polymer (Flory 1940). Similarly, Tejedor et al. tested 12 commonly studied protein condensates by molecular dynamic simulations and demonstrated that the viscosity of condensates is positively correlated with the length of the protein (Tejedor et al. 2023). For a fixed protein sequence, the order of the amino acid can strongly influence the protein conformation and consequently affect the viscoelasticity of resulting condensates of the protein (Sawle and Ghosh 2015). Sundaravadivelu Devarajan et al. demonstrated that the viscosities of both artificial and natural proteins increase with charge segregation (Sundaravadivelu Devarajan et al. 2024). Similarly, Rizvi et al. found that condensates of wild type LAF-1 RGG are more porous and less viscous compared to condensates with a shuffled sequence that contains blocks of oppositely charged residues (Rizvi et al. 2024).

b Post-translational modifications

Macromolecular interactions with a condensate can be modulated via post-translational modifications (PTM), such as phosphorylation, methylation, acetylation, ubiquitination, lipidation, and SUMOylation by altering charge distribution, hydrophobicity, or protein–protein/protein–RNA binding sites (Hofweber and Dormann 2019; Li et al. 2022; Zhang et al. 2024c).

Methylation: Methylation typically involves the addition of one or two methyl groups to the side chains of lysine, arginine, or less frequently, on histidine and glutamate. Arg-methylation has been shown to reduce the propensity of LLPS by weakening Arg–aromatic interactions in FUS (Qamar et al. 2018), Ddx4 (Nott et al. 2015), hnRNP-A2 (Ryan et al. 2018). Compared with unmethylated FUS, methylated FUS shows significantly increased mobility within the condensate (Hofweber et al. 2018). In addition, Qamar et al. demonstrated that hypomethylated FUS results in stiffer condensates as compared to wild type FUS (Qamar et al. 2018).

Phosphorylation: Phosphorylation is a prominent regulator of phase separation, occurring primarily on serine residues (about 90%) and less frequently on threonine or tyrosine (around 10%) (Hofweber and Dormann 2019). The addition of a phosphate group introduces two negative charges under physiological pH (Hofweber and Dormann 2019), substantially altering the residue’s physicochemical properties by creating new electrostatic interactions or disrupting aromatic interactions—particularly when tyrosine is modified (Hofweber and Dormann 2019; Li et al. 2022; Monahan et al. 2017; Wang et al. 2014). If phase separation is driven by electrostatic attraction, additional negative charges via phosphorylation can promote electrostatic attraction or repulsion. In contrast, if aromatic π–π or cation–π interactions (e.g., Tyr/Phe with Arg) are crucial, phosphorylation can weaken these interactions and thus inhibit condensation (Wang et al. 2018b). In support of these dual roles, a phosphomimetic S48E mutation in the TDP-43 N-terminal domain reduces LLPS of TDP-43 in vitro and yields less viscous nuclear assemblies compared to wild-type TDP-43 (Wang et al. 2018a). Similarly, phosphorylation or phosphomimetic substitutions in FUS reduce phase separation and prevent the subsequent liquid-to-solid transition and fibril formation (Monahan et al. 2017). Furthermore, phosphorylation of the SARS-CoV-2 nucleocapsid protein lowers condensate viscosity (Favetta et al. 2024). In contrast, serine phosphorylation in the microtubule-binding domain of Tau by MARK2 kinase promotes its phase separation, presumably through increased electrostatic attraction (Ambadipudi et al. 2017). Consistent with this, hyperphosphorylated Tau is commonly observed in the form of solid aggregates in patients with Alzheimer’s disease and frontotemporal dementia (Kalyaanamoorthy et al. 2024).

Acetylation: Acetylation, typically involves adding an acetyl group to the ε-amino of lysine (Ree et al. 2018). By neutralizing Lys’s positive charge, acetylation reduces electrostatic interactions with negatively charged partners (like DNA/RNA or acidic protein regions) and can disrupt salt bridges. Consequently, acetylation often weakens condensate formation that relies on multivalent electrostatic interactions (Ferreon et al. 2018; Li et al. 2022). For example, acetylation on K311/375 inhibits Tau aggregation and phase separation (Ukmar-Godec et al. 2019). However, in FUS, N-terminal acetylation slightly promoted phase separation but strongly suppressed irreversible aggregation (Bock et al. 2021).

iii. Macromolecular clients

The partitioning of clients to condensates, as an indicator of intramolecular interactions (Qian et al. 2022), can modulate the condensate viscoelasticity. For many RNA binding proteins (e.g., FUS, hnRNP, TDP43), the length, concentration, charge, and structures of their client RNA can strongly modulate condensate viscoelasticity (Guo and Shorter 2015). RNAs typically increase condensate viscosity, for example, CLN3 and BNI1 RNA increase the viscosity of Whi3 condensates in a concentration-dependent manner (Zhang et al. 2015). Similarly, RNA strongly increases the viscosity of SARS-CoV-2 Nucleocapsid protein condensates from ~ 10 Pa s to ~ 400 Pa s, as shown in Fig. 1 (Favetta et al. 2024). Cation-π and π-π interactions between protein and RNA can increase the viscoelasticity of [RGRGG]_5_ condensates (Alshareedah et al. 2021b). An exception was observed in LAF-1 condensates, where increasing the concentration of PolyU_50_ lowered condensate viscosity, potentially due to RNA induced disturbance of IDR-IDR interactions (Elbaum-Garfinkle et al. 2015).

The viscosity of RNA-binding condensate can be altered by the length of RNAs. Wei et al. showed adding short RNA (poly-rA30 and poly-rA15) decreases the viscosity of LAF-1 condensate, while adding long RNA (poly-rA3k) increased condensate viscosity (Wei et al. 2017). Chou et al. showed that increasing RNA length can slow down the dynamics of FUS condensates (Chou and Aksimentiev 2024). Similarly, Keenen et al. suggest that increasing the length of DNA can increase the viscosity of HP1α-DNA condensates (Keenen et al. 2021). Compared to linear RNAs, RNAs that have a secondary structure have been observed to more strongly increase the viscoelasticity of [RGPGG]_5_ condensate (Fig. 1) (Alshareedah et al. 2021b).

In multi-component protein condensates, client proteins can significantly alter the viscoelastic properties of condensates (Banani et al. 2016). These client proteins may strengthen or introduce new contacts, thus increasing the viscoelasticity of condensates. Alternatively, clients can compete with scaffold proteins for binding sites, resulting in reduced condensate viscoelasticity or even dispersion of the condensate. For example, Wang et al. demonstrated that both α-synuclein and synaptic vesicles (SVs), two clients for synapsin condensates, can increase the viscosity of synapsin condensates by ~ 100-fold in vitro (Fig. 1). The effect of α-synuclein is even more dramatic in live cells, regulating the viscoelasticity of synapsin condensates by 10,000-fold (Fig. 1). In contrast, Bouchard et al. demonstrated that increasing the concentration of DAXX in SPOP/DAXX condensates leads to a more liquid-like condensate state (Bouchard et al. 2018). Guo et al. showed that Karyopherin-β2 can inhibit and reverse the fibrillization of FUS, TAF15, EWSR1, hnRNPA1, and hnRNPA2 (Guo et al. 2018), highlighting how client proteins can counteract pathological transitions of the condensate.

iv. Environmental factors

Concentration of biomolecules and crowding reagents: Literature suggests a strong positive correlation between condensate viscosity and the density of biomolecules inside the condensate (Tejedor et al. 2023). In simple single-component protein condensates, altering bulk protein concentration changes the volume fraction but not the concentration of the condensed phase. Thus, the viscosity of these condensates is expected to remain constant. However, in multi-component systems such as PEG-driven protein condensates, increasing PEG concentration can elevate condensate viscosity by reinforcing protein–protein interactions through crowder-mediated depletion (Das et al. 2024a; Kaur et al. 2019; Marenduzzo et al. 2006; Wang et al. 2024). For example, Wang et al. showed that increasing the concentration of PEG from 3 to 10% can increase the viscosity of synapsin condensates from 25 to 115 Pa s.

Salt concentration: Increasing salt concentration primarily influences condensates driven by electrostatic interactions through charge screening. However, salt concentration can also modulate other types of forces, such as cation-$\pi$ and dipole–dipole that drive phase separation (Brangwynne et al. 2015). The reentrant phase separation of some proteins also suggests the influence of salt can be more complex than simply screening charge (Krainer et al. 2021). The viscoelasticity of most proteins reported in the literature show a negative correlation with salt concentration (Fig. 1). In these condensates, increasing salt will weaken electrostatic attraction between molecules and eventually dissolve the condensate. For instance, the viscosity of LAF-1 condensates decreases from 34 to 8 Pa s when NaCl concentration was increased from 125 to 400 mM (Elbaum-Garfinkle et al. 2015). Similar salt effect was observed for condensates of glycinin (Mangiarotti et al. 2023), PolyK + heparin (pk:H) (Ghosh et al. 2021), and PGL-3 (Jawerth et al. 2020; Jawerth et al. 2018), as plotted in Fig. 1. The effect of salt can be more complicated during reentrant phase separation. For instance, the viscoelasticity of HA-Protamine condensates under 4 M NaCl is higher than that under 0 M NaCl, with intermediate concentrations of NaCl inhibiting condensate formation (Table 2) (Hong et al. 2022).

Temperature: The viscosity of a polymer solution typically decreases with temperature, scaling with ${e}^\frac{1}{T}$ (Steinfeld et al. 1999). Similarly, for condensates that have an upper critical solution temperature (UCST), increasing temperature will make molecules in the condensate more dynamic, thus reducing condensate viscosity (Alshareedah et al. 2024b). While for lower critical solution temperature (LCST) proteins, increasing temperature may strengthen molecular interactions and harden the condensate (Vidal Ceballos et al. 2022).

Other factors: It has been demonstrated that fixation can alter the appearance of condensates in cells (Irgen-Gioro et al. 2022; Miné-Hattab 2023). Schneider et al. showed that the fixation of GFP–RBM20^R636S^ condensate strongly slows the dynamics of RBM20 inside the condensate (Schneider et al. 2020). On a separate note, Shen et al. demonstrated that shear stress on FUS and several other protein condensates can trigger $\beta$-sheet formation in the condensate, resulting in a liquid-to-solid transition of the condensate (Shen et al. 2020).

e Cellular activities

The properties of biomolecular condensates can also be regulated by active cellular processes. Studies have shown that ATP depletion dramatically changes the physical properties of phase-separated structures (Brangwynne et al. 2011; Feric et al. 2016). For instance, under ATP-depleted conditions, nucleoli exhibit slower fusion speeds and reduced fibrillarin (FIB1) dynamics. Increasing the enzymatic ATPase activity of Dhh1 slows down the aging of these condensates (Linsenmeier et al. 2022). Likewise, SGs show slower FRAP recovery following ATP depletion, indicating a higher condensate viscosity (Jain et al. 2016). In the same study, they also showed that upon oxidative stress, SGs assemble in cells with their viscosity positively correlated with the stress level.

f Time/maturation.

Although many early studies of intracellular phase separation focused on condensates as purely liquid-like droplets with rapid internal molecular exchange, it has become increasingly clear that some of these condensates can exhibit a continuous change of physical states over time (Alshareedah et al. 2024a; Jawerth et al. 2020), often accompanied with an increase of condensate viscosity. Several proteins will eventually form an ordered structure, such as fibrils (Emmanouilidis et al. 2024; Patel et al. 2015), while other proteins can still maintain a disordered structure, such as glassy liquid or amorphous solid (Alshareedah et al. 2024a; Jawerth et al. 2020; Tejedor et al. 2023). Patel et al. showed that FUS transitions from a liquid to a fibrillar state, accelerated by disease-associated mutations (Patel et al. 2015). Jawerth et al. reported that changes in condensate material properties over time indicates condensates do not age into a gel. Instead, condensates behave like Maxwell fluids, in which viscosity increases over time while elasticity remains largely unchanged. In contrast, Alshareedah et al. reported A1-LCD ages into non-fibrillar, β-sheet-containing, semi-crystalline elastic solids (Alshareedah et al. 2024a).

Overall, factors that promote phase separation tend to also increase condensate viscoelasticity (Rekhi et al. 2024). This observation aligns with the principle that viscosity reflects the strength of molecular interactions and that increased interactions enhance a system’s propensity for phase separation. By understanding the emergence and regulation of condensate viscoelasticity, researchers can unravel the fundamental principles of intracellular organization and conditions under which condensates malfunction.

g Other bulk properties of biomolecular condensates.

The formation of biomolecular condensates provides a special chemical environment that distinguishes them from the surrounding dilute phase. Several studies suggest that the unique chemical environment of a condensate allows selective recruitment or depletion of molecules, which is particularly crucial for condensate targeted drug design (Basu et al. 2023; Dai et al. 2024a; Kilgore et al. 2024; Testa et al. 2021). A wide range of condensates has been shown to create a highly hydrophobic environment that allows the enrichment of lipids (Dumelie et al. 2024). The accumulation of net charge of IDR in the nucleus sets up a pH gradient, which is a potential regulator for enzyme activities in the nucleus (King et al. 2024). The chemical environment of condensate may also be age-related. Yu et al. demonstrated an age-dependent pH gradient within condensates (Yu, W. et al. 2025). Moreover, the chemical environment difference between condensate and the surroundings can generate forces that push the condensate toward solvent conditions that favor condensate dissolution (Jambon-Puillet et al. 2024).

In studies involving condensate viscoelasticity, the role of condensates in the formation of pathological fibrils is a key area of investigation. Increases in condensate viscoelasticity are often correlated with the formation of solid aggregates. However, it remains unclear whether condensates promote or inhibit fibril assembly. Two possible pathways have been proposed. On the one hand, condensates have been shown to facilitate fibril formation by concentrating amyloidogenic molecules, thereby accelerating fibril assembly (Babinchak et al. 2019; Molliex et al. 2015; Piroska et al. 2023; Sahu and Ross 2023; Wegmann et al. 2018). Additionally, they can promote conformational changes within disordered regions of the condensate-forming protein, leading to the formation of amyloid-like structures (Emmanouilidis et al. 2024; Ray et al. 2020). On the other hand, the sequestration of molecules into condensates may prevent them from going towards the fibril-formation pathway (Küffner et al. 2021). In this scenario, condensates act protectively by maintaining a low concentration of monomers in the dilute phase, thereby reducing fibril formation (Das et al. 2024b; Lipiński et al. 2022). Beyond the bulk viscoelasticity of condensates, several recent studies have highlighted a unique role of the condensate interface in regulating the formation of fibrils (Choi et al. 2024; Dai et al. 2023; Das et al. 2024b; Emmanouilidis et al. 2024; Farag et al. 2022; Linsenmeier et al. 2023; Lipiński et al. 2022; Shen et al. 2023).

### The interfacial tension of biomolecular condensates

Biomolecular condensates have a well-defined boundary that separates them from the surrounding environment. The imbalance of molecular forces at the condensate interface leads to an interfacial tension, driving processes that minimize the area of interfaces such as condensate fusion. The interfacial tension of condensates has been reported to regulate many processes where condensates interact with intracellular structures such as DNAs (Strom et al. 2024), microtubules (Setru et al. 2021), autophagosomes (Agudo-Canalejo et al. 2021; Yamasaki et al. 2020), endocytic pits (Bergeron-Sandoval et al. 2021), and nucleolar layers (Feric et al. 2016; Fisher & Elbaum-Garfinkle 2020; Yu et al. 2021).

Several methods have been developed to study the interfacial tension of condensates, including condensate fusion-based capillary velocity measurements (Eggers et al. 1999; Ghosh and Zhou 2020; Ghosh et al. 2021; Sahu and Ross 2023), surface fluctuation spectrum (Caragine et al. 2018; Law et al. 2023), sessile drop assays (Feric et al. 2016; Holland et al. 2023), micropipette aspiration (Mohammadi et al. 2018; Roggeveen et al. 2023; Shen et al. 2023; Wang et al. 2021b, 2024; Wang et al. 2022a), and optical tweezers-based condensate deformation (Jawerth et al. 2018; Zhou, H. 2020). Among these techniques, condensate fusion analysis is the most commonly used method in the literature. It provides a ratio of viscosity to interfacial tension, necessitating a separate viscosity measurement to determine the condensate’s interfacial tension. The presence of condensate elasticity further complicates the extraction of interfacial tension from measured capillary velocity (Ghosh et al. 2021; Style et al. 2017). In contrast, micropipette aspiration can directly measure the interfacial tension and viscoelasticity of condensates in one experiment.

Here, we summarize recent studies on the interfacial tension of condensates (Fig. 2), review how condensate interfaces regulate various cellular structures and activities, discuss factors that regulate condensate interfacial tension, and highlight additional interfacial properties of these systems.

### Cellular activities regulated by the interfacial tension of condensates

a Condensate formation and size control

The nucleation of a liquid condensate is governed by the interfacial tension between the droplet and the surrounding environment (Kashchiev 2000; Shimobayashi et al. 2021). Both theoretical and experimental evidence indicate that, in heterogeneous systems such as the intracellular space, the emergence and growth of condensates depend on a balance between local molecular concentrations and the condensate’s interfacial tension.

To minimize the total area of interfaces, smaller condensates tend to coalesce into one large droplet. This can be achieved by either condensate coalescence (Eggers et al. 1999; Elbaum-Garfinkle et al. 2015) or Ostwald ripening, in which smaller condensates dissolve due to higher internal pressure, leaving their constituent molecules to diffuse into larger condensates (Adamson and Gast 1967; Bressloff 2020; Meng et al. 2024). The elastic stiffness of the intracellular space adds a barrier to condensate expansion, resulting in a suppression of the formation and growth of condensates (Banerjee et al. 2024; Meng et al. 2024; Rosowski et al. 2020; Style et al. 2018).

b Multiphase condensates and condensate miscibility

Several condensates, such as the nucleolus (Feric et al. 2016; Riback et al. 2023), exhibit multiphase organization, where phases with distinct compositions coexist in the same droplet or form demixed droplets, determined by the interfacial tensions between each phase (Gouveia et al. 2022). The structural organization of a multiphase condensate is essential for its function. In the three-layered nucleolus, rDNA transcription occurs in the innermost layer, rRNA processing in the middle layer, and ribosome assembly in the outer layer, after which ribosomes can be transported to the surrounding nucleoplasm (Tiku and Antebi 2018). Enzymes required for these processes need to be selectively localized to each layer to facilitate the respective biomolecular processes. In neurons, excitatory and inhibitory postsynaptic densities (e/iPSDs) were separated into distinct condensates, even within a single dendritic spine (Zhu et al. 2024). This demixing of condensate phases ensures that excitatory and inhibitory signals remain confined to discrete compartments, which is crucial for proper neuronal function. By modulating the interfacial tension between different phases through altering protein or RNA concentration, cells can dynamically tune the miscibility and architecture of condensate phases (Fabrini et al. 2024; Kaur et al. 2021; Pei et al. 2024b). Altering the interfacial tension of condensates can also lead to the loss of key cellular functions. For example, Bouchard et al. demonstrated that mutations in the tumor suppressor SPOP disrupt its colocalization with death-domain-associated protein (DAXX), thereby impairing related enzymatic activities (Bouchard et al. 2018).

iii. Interaction between condensates and other cellular structures

Although the interfacial tension of condensates is relatively small (10^−4^ ~ 10^−7^ N/m) compared to that of oil-like droplets (Fig. 2) (Wang et al. 2021b), forces generated by the interface of a condensate can be comparable to those provided by molecular motors (Jiang et al. 2003; Lecuit et al. 2011; Valentine et al. 2006).

Wetting between condensates and solid surfaces reflects a competition between the interfacial tension of condensates that promotes dewetting and the adhesion between condensate and the solid surface. Growing evidence indicates that the wetting between condensates and cellular structures such as membranes, the cytoskeleton, and the genome, plays a critical role in mediating their biological function (Gouveia et al. 2022).

#### Membranes

The wetting of condensates on cellular membranes directly affects processes such as autophagy, endocytosis, and synaptic vesicle clustering (Agudo-Canalejo et al. 2021; Mangiarotti et al. 2023; Mangiarotti and Dimova 2024; Milovanovic et al. 2018; Snead et al. 2022). During autophagy, the extent of condensate wetting on the autophagosome determines whether the condensate can be fully enveloped and subsequently cleared (Agudo-Canalejo et al. 2021). Similarly, the elongation of ZO-1 condensates is essential for the formation of tight junctions (Pombo-García et al. 2024). Condensates can also alter the curvature of the wetted membrane, potentially initiating membrane remodeling events (Banjade and Rosen 2014; Mangiarotti et al. 2023; Mangiarotti and Dimova 2024; Mangiarotti et al. 2025), such as endocytosis or vesicle formation (Mondal and Baumgart 2023). Bergeron-Sandoval et al. demonstrated that the mechanical stress stored by endocytic protein condensates can remodel the membrane and facilitate endocytosis (Bergeron-Sandoval et al. 2021).

Below the elastocapillary length (*γ/E*), interfacial tension dominates over the bulk elasticity of a condensate. As an elegant example that demonstrates the mechanical stiffness that arises from the interfacial tension of condensates, lipid droplets (interfacial tension around 40 mN/m) have been shown to deform the nuclear envelope and act as a mechanical stressor to the nucleus, potentially facilitating cancer development (Ivanovska et al. 2023; Loneker et al. 2023). This example further underscores the importance of interfacial tension at small length scales. Although bulk oil behaves as a pure liquid at the macroscopic scale, a micrometer-sized oil droplet behaves almost like a rigid particle because the stresses required to deform the oil-water interface are prohibitively large.

Some protein condensates can exhibit a lipid selectivity that initiates and stabilizes phase separation within the membrane. The LAT–Grb2–Sos1 condensate, essential for T cell activation, exemplifies this coupling by promoting raft-like liquid-ordered domains in the plasma membrane (Wang et al. 2022a). In the other way around, interactions with the membrane can nucleate and promote protein condensation while constraining their ultimate size (Shelby et al. 2023; Snead et al. 2022; Wang et al. 2023). For instance, the presence of membranes markedly lowers the phase separation concentration of Whi3.

#### Cytoskeletons

The cytoskeleton, composed primarily of actin filaments, microtubules, and intermediate filaments, is a dynamic network that provides structure, organization, and mechanical force within the cell (Lodish 2008; Zhao et al. 2021). It has been reported that the upstream activator of actin, actin regulators (nephrin/NCK/N-WASP), and actin polymerase (VASP) can form condensates to concentrate actin monomers, nucleate actin filaments, and modulate actin polymerization (Banjade and Rosen 2014; Su et al. 2016; Walker et al. 2025). The growth of actin bundles out of these condensates reflects a competition between the bending energy of actin filaments and the interfacial tension of condensates (Chandrasekaran et al. 2024; Graham et al. 2023). A similar phenomenon is observed regarding the polymerization of microtubules in mitotic centrosomes (Enos et al. 2018; Mittasch et al. 2020).

Condensates that fully wet and form a layer on cytoskeletal filaments can become unstable and transit into a string of regularly spaced droplets, driven by the interfacial tension of the condensate. This instability transition can lead to contraction of actin bundles (Weirich et al. 2017), facilitate microtubule branching (Setru et al. 2021), and can be regulated by molecular modifications such as the phosphorylation of tau (Hernández-Vega et al. 2017).

More generally, the interface between condensates and the surrounding cytoplasm can be affected by the physical, biochemical, and mechanical interactions with cytoskeletal components (Böddeker et al. 2022; Chandrasekaran et al. 2024; Feric and Brangwynne 2013; Gouveia et al. 2022; Shin and Brangwynne 2017; Su et al. 2016; Walker et al. 2025). It has been shown that the cytoskeleton and nuclear actin network can regulate the size of biomolecular condensates through mechanisms beyond Ostwald ripening. For instance, in large cells such as oocytes, the nuclear actin network counteracts gravity-driven motion and coalescence of nucleoli, maintaining their spatial organization (Feric and Brangwynne 2013; Feric et al. 2015). Additionally, cytoskeletal dynamics actively promote the fusion and fission of condensates. Microtubules, for example, accelerate stress granule assembly by facilitating condensate coalescence through pushing, pulling, or sliding of condensates along microtubule tracks (Chernov et al. 2009).

Condensates can also modulate the surrounding cytoskeleton. For instance, stress granules lead to a compaction of the surrounding microtubule network (Böddeker et al. 2022), potentially consistent with microtubule’s role in accelerating the assembly of stress granules (Chernov et al. 2009).

#### DNAs and RNAs

A number of studies have shown that the regulation of gene expression involves condensate formation (Gibson et al. 2019; Guo et al. 2019; Hallegger et al. 2021; Lu et al. 2018; Pei et al. 2024a; Shin et al. 2018; Wei et al. 2020). Similar to how membrane-binding proteins can phase separate at lower concentrations (Hsu et al. 2023), DNAs have been shown to reduce the saturation concentration (*C*_sat_) needed for phase separation of Klf4 (Morin et al. 2022). Forces generated by condensates can aid DNA remodeling. It has been shown that FoxA1 condensate can generate forces of about 0.4–0.6 pN on a single λ-phage DNA molecule (length 6 or 8 μm) (Quail et al. 2021). A similar phenomenon was reported for HP1 (Keenen et al. 2021). Using this principle, Shin et al. developed CasDrop, which can trigger condensate formation at targeted genomic loci (Shin et al. 2018; Strom et al. 2017).

## Factors that regulate the interfacial tension of condensates

From the Flory–Huggins theory, the interfacial tension of condensates is determined by $\chi /{a}^{2}$, where $\chi$ is the driving force of phase separation, and $a$ is the size of phase separating molecules (Berry et al. 2018; Dill and Bromberg 2010). Consequently, factors that influence the driving force of phase separation or the molecular size should, in principle, also affect the interfacial tension of the resulting condensates (Dignon et al. 2020; Wang et al. 2018b). However, as shown in Fig. 2, the interfacial tension and viscosity of a biomolecular condensate appear to be entirely independent properties that can be regulated separately. This is in contrast to the apparent correlation and co-regulation of the elasticity and viscosity of condensates observed in the literature (Fig. 1).

a Sequence and composition

The molecular sequence of a protein is a primary determinant of the interfacial tension of the resulting condensates. Modulations to protein sequence often lead to co-regulation of the interfacial tension and viscosity of a condensate. For example, Pyo et al. demonstrated that increasing polymer length elevates droplet interfacial tension (Pyo et al. 2023), mirroring the sequence-dependent effects on condensate viscosity reported by Tejedor et al. (2023). Similarly, increasing the length of RNA in RGG-PolyU condensate can also increase the condensate interfacial tension (Laghmach et al., 2022). Moreover, simulations by Sundaravadivelu Devarajan et al. indicate that charge segregation affects both the viscosity and interfacial tension of E-K, LAF1, and DDX4 condensates (Sundaravadivelu Devarajan et al. 2024).

Independent regulation of the interfacial tension and viscosity of a condensate can be achieved via compositional modulations. Wang et al showed that SVs, α-synuclein, and the crowding reagent PEG all increase the viscosity of synapsin condensates. However, these factors have distinct effects on the interfacial tension of synapsin condensates: the interfacial tension increases with PEG, decreases with SVs, but remains unaltered with the partitioning of α-synuclein (Fig. 2) (Wang et al. 2024).

Alterations in the phase structure or miscibility of condensates are linked to changes in the interfacial tension between phases. To elucidate the underlying molecular grammar, Pei et al. compared various combinations of intrinsically disordered regions (IDRs) and demonstrated that IDRs enriched in serine or aromatic amino acids tend to form miscible condensates, whereas IDRs with high charge levels form immiscible condensates even when serine levels are artificially elevated (Pei et al. 2024b). Rana et al. showed that increasing the oligomerization state of an intrinsically disordered protein enhances immiscibility and promotes multi-phase formation from a homogeneous condensate (Rana et al. 2024). Notably, chemically active condensates can regulate their interfacial tension by modifying molecular interactions at their interface. This self-regulation enables condensate growth and division, making them potential models for protocells (Nakashima et al. 2021; Zwicker et al. 2017).

b Environmental factors and cellular activities

Several environmental factors can co-regulate the interfacial tension and viscosity of condensates. As shown in Fig. 2, increasing salt concentration decreases both the viscosity and interfacial tension of several distinct types of condensates, including Glycinin (Mangiarotti et al. 2023), PGL-3 (Jawerth et al. 2018), and [RGRGG]_5_-dT40 (Alshareedah et al. 2021c), resulting in modifications of condensate-membrane wetting behavior (Mangiarotti et al. 2023) or changes in the structure of multiphase condensates (Erkamp et al. 2024). Enzymatic activities may also regulate the interfacial tension of condensates. For instance, calcium/calmodulin-dependent protein kinase II (CaMKII), a key kinase for neuronal function (Yasuda et al. 2022), can disperse synapsin and liposome clusters in vitro (Milovanovic et al. 2018). However, the activation of CaMKII was reported to promote the phase separation of GluN2B (Hosokawa et al. 2021), and regulate the multiphasic structure of the postsynaptic density (Yamada et al. 2025; Zeng et al. 2019).

iii. Surfactants and Pickering agents

Amphiphilic biomolecules can localize to the interface of condensates and reduce their apparent interfacial tension (Folkmann et al. 2021; Kelley et al. 2021; Sanchez-Burgos et al. 2023; Welsh et al. 2022). These surfactant-like molecules regulate condensate size and inhibit fusion events, functioning analogously to lipid bilayers in membrane-bound organelles. Several key examples illustrate this regulatory mechanism in cellular contexts. The protein Ki-67 localizes to the surface of mitotic chromosomes and stabilizes chromosome clustering (Cuylen-Haering et al. 2020). Similarly, Atg19 in its normal state localizes to the surface of Ape1 condensates, regulating selective autophagy via modulated condensate-membrane interactions (Yamasaki et al. 2020). The protein NO145 localizes to the outer surface of *Xenopus laevis* nucleoli and could contribute to the size control of nucleolus (Brangwynne et al. 2011). Consistent with this principle, Kelley et al. engineered several amphiphilic proteins that function as surfactants, localizing to the surface of LAF-1 RGG condensates and reduce condensate size (Kelley et al. 2021).

In addition to forming a layer at the surface of condensates, some biomolecules assemble into discrete clusters, analogous to solid particles that can stabilize emulsions (Abkarian et al. 2007). Folkmann et al. demonstrated that MEG-3 forms clusters on the surface of P granules, acting as a pickering agent that decelerates condensate coarsening (Folkmann et al. 2021). This stabilization of P granules is critical to their asymmetric enrichment during zygote polarization. In a synthetic biology approach, Oh et al. engineered several solid “protein cages” that localize to the surface of condensates (Oh et al. 2025).

## Other important interfacial properties

In addition to interfacial tension, another emerging critical feature of condensate interfaces is their interfacial electric/chemical potential (Dai et al. 2024a, 2024b). The unique chemical environment within condensates, which differs markedly from the surrounding dilute phase, can generate a steep electrochemical potential gradient across the interface. These gradients enable condensates to recruit molecules or ions to balance their interfacial charge (Folkmann et al. 2021; Majee et al. 2024), leading to the formation of an electric double layer. As a result, there is a significant electrochemical potential that can catalyze redox reactions at the interface of condensates (Dai et al. 2023; Guo et al. 2024).

What is the level of interfacial electric/chemical potential? Zeta potential, which is the electrical potential at the edge of the interfacial double layer around the surface of charged particle, is a potential indicator. From Welsh et al., the condensate with higher zeta potential showed lower propensity to fuse, which indicates the interfacial double layer is essential for condensate stability (Welsh et al. 2022). Dai et al. demonstrated that the pH difference of a resilin-like polypeptide condensate leads to a 47 mV interfacial potential, enabling condensates to spontaneously generate reactive oxygen species (ROS), both in vitro and in cells. It is known that ROS is highly involved in promoting protein aggregation (Squier 2001). The interfacial potential may underlie condensate interface’s ability to promote protein aggregation (Chen et al. 2025; Choi et al. 2024; Emmanouilidis et al. 2024; Linsenmeier et al. 2023; Lipiński et al. 2023; Sahu et al. 2025; Shen et al. 2023). When contacting other cellular compartments such as membranes, condensates can produce a localized membrane potential that might be relevant to neuronal signaling(Gurunian et al. 2024).

The interface of a condensate can also act as a barrier that resists mass transport and screen fluctuations from the environment (Bhatia and Dutta 2023; Taylor et al. 2019). Zhang et al. proposed a mechanism for this barrier: when a molecule in the dilute phase diffuses to the condensate interface, it is not immediately absorbed. Instead, it often bounces off, leading to effective interfacial resistance (Zhang et al. 2024b).

## Conclusions and outlook

In this review, we discuss how condensate viscoelasticity and interfacial tension are involved in cellular functions. We also highlight how abnormal condensate material properties can lead to various diseases, including cancer, neurodegenerative disorders, and viral infections. As a resource to the community, we summarize quantitative measurements of condensate viscoelasticity and interfacial tension in the literature, and describe how sequence features, client proteins, RNAs, post-translational modifications, environmental factors, and cellular activities regulate these properties.

Future directions should focus on:

1. Development of non-invasive and high throughput tools to study the material properties of condensates in living systems.
2. Exploring and characterizing interfacial properties beyond mechanical tension, such as the electrical and chemical properties of condensate interfaces. It is also important to study how molecular factors regulate these interfacial properties.
3. Studying factors that regulate condensate viscoelasticity in vivo and establishing the relation (or distinction) between a solidified condensate and pathological protein aggregates/fibrils.
4. Establishing relationships between condensate composition, structure, and material properties in vivo.

## Data Availability

No datasets were generated or analysed during the current study.
